# Identifying the predictive role and the related drugs of oxidative stress genes in the hepatocellular carcinoma

**DOI:** 10.1002/cnr2.1978

**Published:** 2024-04-10

**Authors:** Guole Nie, Xingwang Zhu, Honglong Zhang, Haiping Wang, Jun Yan, Xun Li

**Affiliations:** ^1^ The First School of Clinical Medicine Lanzhou University Lanzhou China; ^2^ Department of General Surgery The First Hospital of Lanzhou University Lanzhou China; ^3^ Key Laboratory of Biotherapy and Regenerative Medicine of Gansu Province Lanzhou China

**Keywords:** GEO, hepatocellular carcinoma, overall survival, oxidative stress‐related genes, prognostic, TCGA

## Abstract

**Background and Aims:**

Oncogenesis and tumor development have been related to oxidative stress (OS). The potential diagnostic utility of OS genes in hepatocellular carcinoma (HCC), however, remains uncertain. As a result, this work aimed to create a novel OS related‐genes signature that could be used to predict the survival of HCC patients and to screen OS related‐genes drugs that might be used for HCC treatment.

**Methods:**

We used The Cancer Genome Atlas (TCGA) database and the Gene Expression Omnibus (GEO) database to acquire mRNA expression profiles and clinical data for this research and the GeneCards database to obtain OS related‐genes. Following that, biological functions from Gene Ontology (GO) and the Kyoto Encyclopedia of Genes and Genomes (KEGG) were performed on differentially expressed OS‐related genes (DEOSGs). Subsequently, the prognostic risk signature was constructed based on DEOSGs from the TCGA data that were screened by using univariate cox analysis, and the Least Absolute Shrinkage and Selection Operator (LASSO) regression, and multivariate cox analysis. At the same time, we developed a prognostic nomogram of HCC patients based on risk signature and clinical‐pathological characteristics. The GEO data was used for validation. We used the receiver operating characteristic (ROC) curve, calibration curves, and Kaplan–Meier (KM) survival curves to examine the prediction value of the risk signature and nomogram. Finally, we screened the differentially expressed OS genes related drugs.

**Results:**

We were able to recognize 9 OS genes linked to HCC prognosis. In addition, the KM curve revealed a statistically significant difference in overall survival (OS) between the high‐risk and low‐risk groups. The area under the curve (AUC) shows the independent prognostic value of the risk signature model. Meanwhile, the ROC curves and calibration curves show the strong prognostic power of the nomogram. The top three drugs with negative ratings were ZM‐336372, lestaurtinib, and flunisolide, all of which inversely regulate different OS gene expressions.

**Conclusion:**

Our findings indicate that OS related‐genes have a favorable prognostic value for HCC, which sheds new light on the relationship between oxidative stress and HCC, and suggests potential therapeutic strategies for HCC patients.

## 1 INTRODUCTION

Hepatocellular carcinoma (HCC) is the most common type of primary liver tumor, accounting for more than 90% of all primary liver tumors.^1^ HCC is the fifth most common cause of cancer worldwide and the second leading cause of cancer death and the overall survival rate for HCC remains poor.^2^ Current HCC evaluation and treatment do not satisfy the standards for early identification and longer overall survival. As a result, new biomarkers with higher predictive value must be investigated to improve patient prognosis.

The mechanism of HCC development is still unknown at the moment. HCC is linked to many well‐known causes, including alcohol abuse, chronic HBV or HCV infection, non‐alcoholic fatty liver disease, and exposure to dietary toxins including aflatoxins.^3^ Recent studies have revealed that oxidative stress, through the excessive production of reactive oxygen species (ROS), plays an important role in tumorigenesis and cancer progressiong.^4^, ^5^, ^6^ HCV and HBV infection causes oxidative DNA damage, which actively encourages hepatocellular tumorigenesis.^7^ Therefore, OS may be closely related to the occurrence of HCC. However, the prognostic value of these OS genes in HCC and their associated mechanisms require further investigation.

Current diagnostic methods for HCC are mainly dependent on imaging and histopathological examination. In recent years, large‐scale tumor genome sequencing data has provided an excellent opportunity to identify potential molecular markers.^8^, ^9^ In present study, using mRNA sequencing data and clinical information from The Cancer Genome Atlas (TCGA) and Gene Expression Omnibus (GEO) datasets, we screened OS genes linked with HCC prognosis and developed a risk signature and prognosis‐related nomogram based on prognosis‐related OS genes. In addition, we screened small molecule drugs linked to OS genes to provide a new perspective on HCC treatment.

## 2 MATERIALS AND METHODS

### 2.1 Data collection and identification of differentially expressed OS‐related genes (DEOSGs)

The mRNA expression profile dataset contained 374 HCC samples and 50 normal liver tissues, as well as clinical data from the TCGA database^10^ (https://portal.gdc.cance r.gov/) on March 27, 2022. In addition, we downloaded the clinical and gene expression profiles of 167 HCC patients in the GSE76427 dataset from GEO.^11^ Finally, we extracted the complete clinical data (age, stage, sex, survival time) of 279 patients in TCGA and 114 patients in GEO, respectively.

TCGA data was used as a training group and GEO data was used as a validation group. To identify differentially expressed oxidative stress genes, we obtained 611 OS‐related genes with relevance scores ≥7 from the GeneCards website (https://www.genec ards.org). We finally obtained 544 common OS‐related genes in both datasets. Subsequently, we used the edgeR package^12^ for further processing with the criteria of FDR <0.05 and |Log2FC| ≥ 0.5, concerning previous study criteria.^13^ Ultimately, differentially expressed genes were visualized with the heatmap package and ggplot package in R software.

### 2.2 Gene ontology (GO) and kyoto encyclopedia of genes and genomes (KEGG) analyses

To further clarify the potential functions of differentially expressed genes, GO^14^ and KEGG analysis^15^ were performed using the cluster Profiler package in R software and visualized using the enrichplot and graphics packages.

### 2.3 Identification of prognosis‐related DEOSGs and construction of prognostic model

We firstly performed univariate Cox regression and Kaplan–Meier (KM) analysis using the survival R package to identify OS‐related genes from TCGA that were associated with overall survival (OS). Only *p* value <.05 genes were identified as prognosis‐associated DEOSGs in both investigations. To narrow down the spectrum of prognosis‐associated genes, LASSO regression analysis of the above prognosis‐associated DEOSGs was conducted using the glmnet package.^16^ Subsequently, a multivariate cox regression analysis was performed using the survival package (https://cran.r-proje ct.org/packa ge = survival) to create an OS‐related risk signature based on the regression coefficients from the analysis and the expression levels of the selected OS‐related genes. The formula was calculated as follows: risk score = Σexpgene_i_*β_i_, where expgene represents the relative expression value of OS genes and *β* represents the regression coefficient.

The median risk score was used to divide patients into high‐risk and low‐risk groups, and overall survival was compared between the two categories using the survival package's Kaplan–Meier approach and the log‐rank test. To validate the predicted accuracy and ability of the risk score, the survival ROC and time ROC packages^17^ in R software were used. The link between clinical features, risk scores, and prognosis was further investigated using univariate and multivariate Cox regression analyses. Finally, the rms package was used to create a nomogram containing calibration plots to estimate the prognosis of HCC patients. The GEO cohort was used to test the model's prognostic performance.

### 2.4 Identification of potential therapeutic drugs

The DEOSGs have been uploaded to the L1000FWD website (https://maayanlab.cloud/L1000FWD). After that, a table of prospective medications was obtained. We further used PubChem (pubchem.ncbi.nlm.nih.gov) to visualize the above results.

## 3 STATISTICAL METHODS

*R* software (version 4.3.1) was used as a statistical analysis tool in this study. Quantitative data were expressed as mean ± standard error (SEM) or standard deviation (SD). Statistical significance was defined as *p* < .05. Figures were constructed by R software.

## 4 RESULTS

### 4.1 Identification and functional enrichment analysis of DEOSGs

**FIGURE 1:**
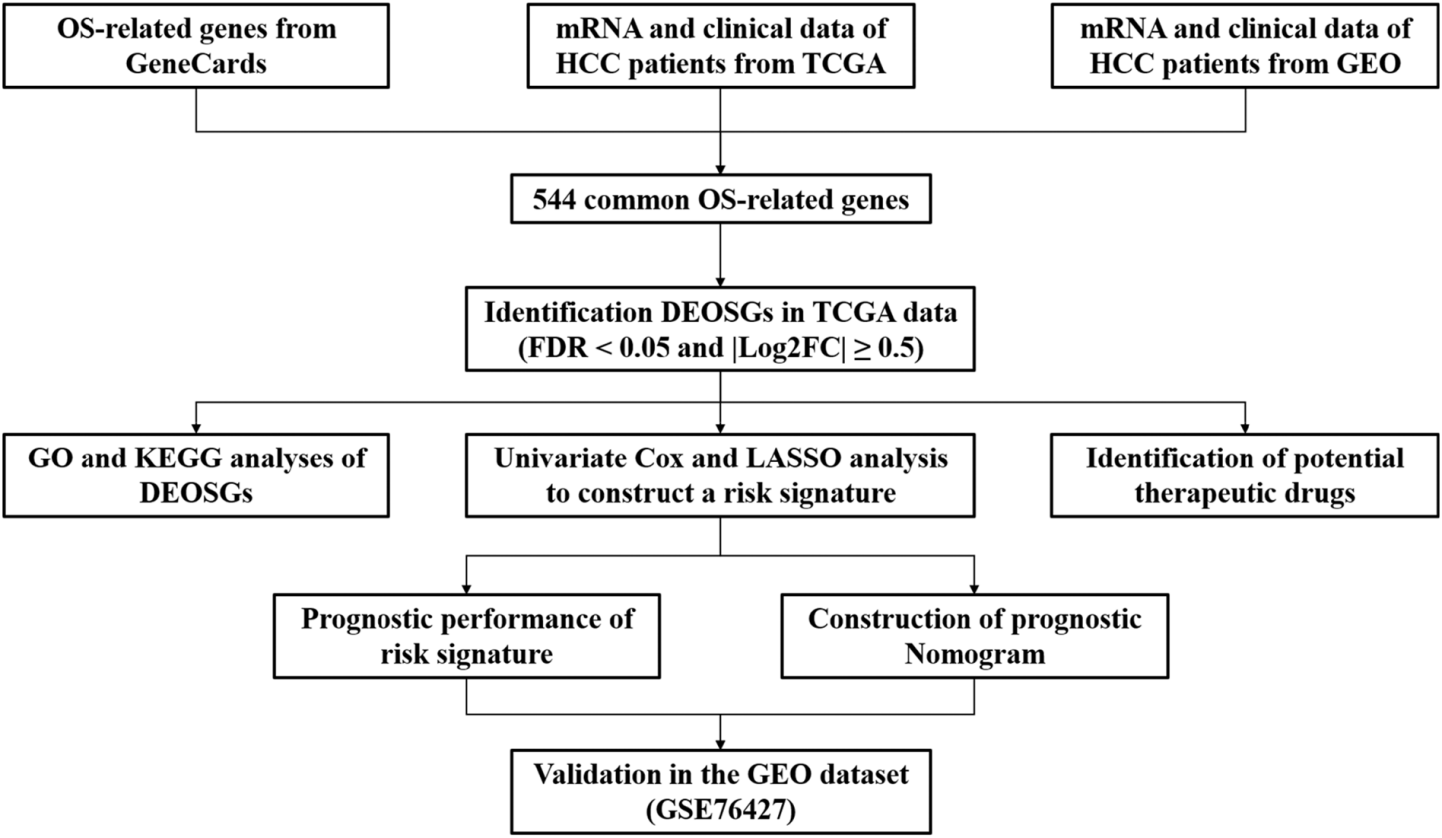
Flowchart of the research.

**FIGURE 2:**
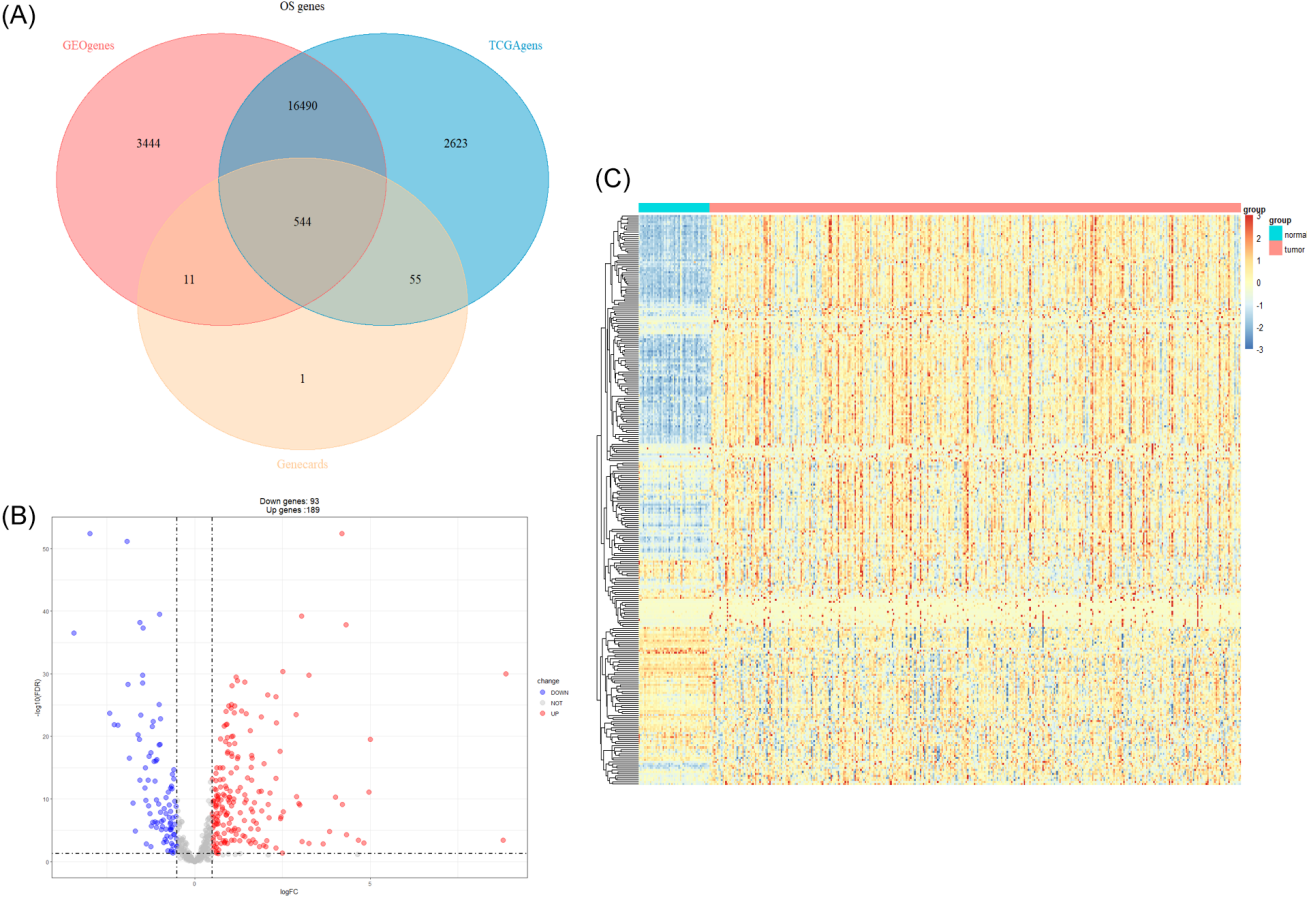
Identification of DEOSGs. (A) The common OS‐related genes in GEO, TCGA, and GeneCards. (B) Volcano plot of DEOSGs between normal and tumor tissues from TCGA. (C) Heatmap of DEOSGs.

**FIGURE 3:**
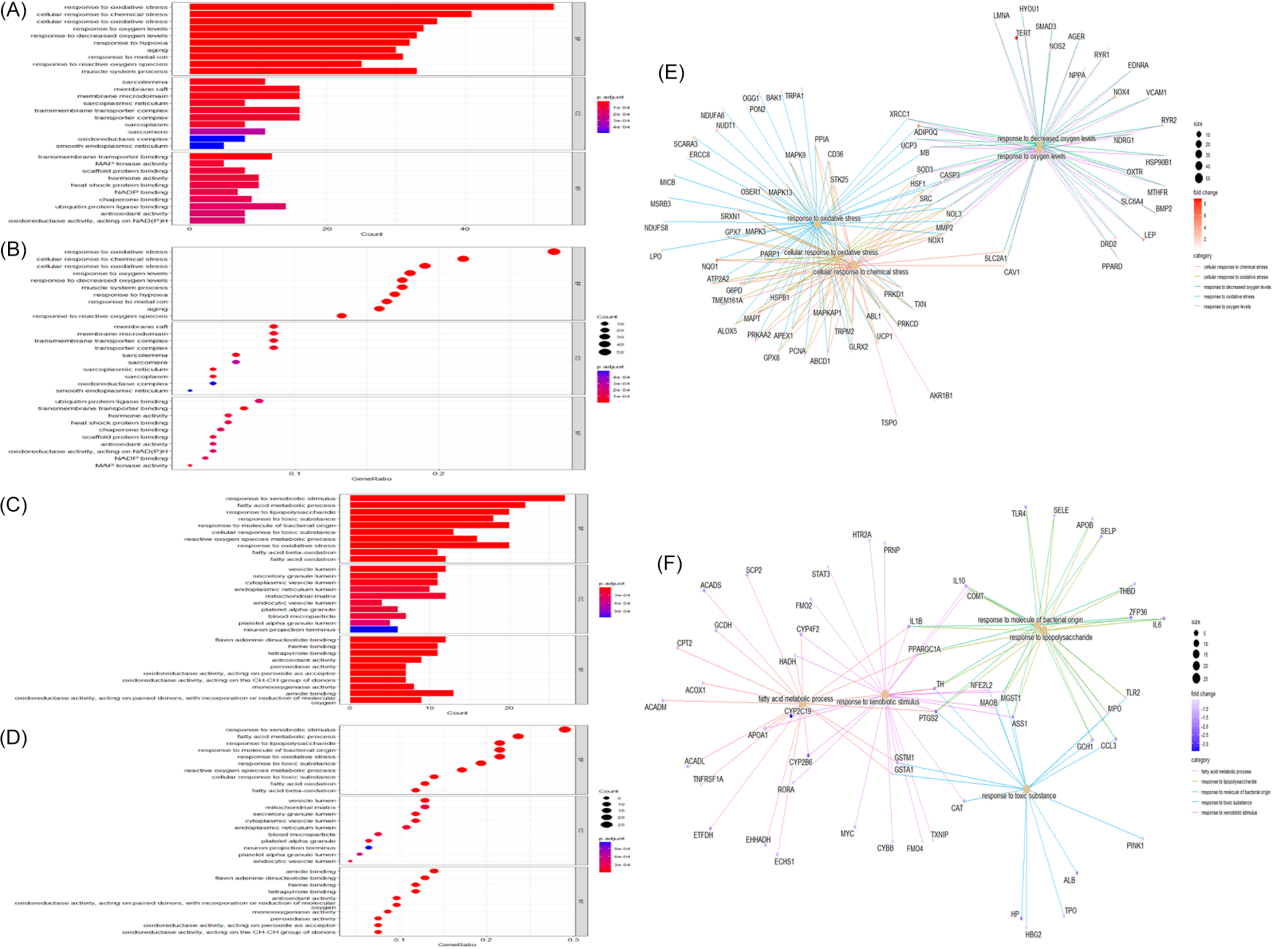
GO analysis of upregulated and downregulated DEOSGs. (A, B) Top 10 classes of GO enrichment terms of upregulated DEOSGs in biological process (BP), cellular component (CC), and molecular function (MF). (C, D) Top 10 classes of GO enrichment terms of downregulated DEOSGs in biological process (BP), cellular component (CC), and molecular function (MF). (E) Grid diagram of top 5 GO terms and upregulated DEOSGs. (F) Grid diagram of top 5 GO terms and downregulated DEOSGs.

**FIGURE 4:**
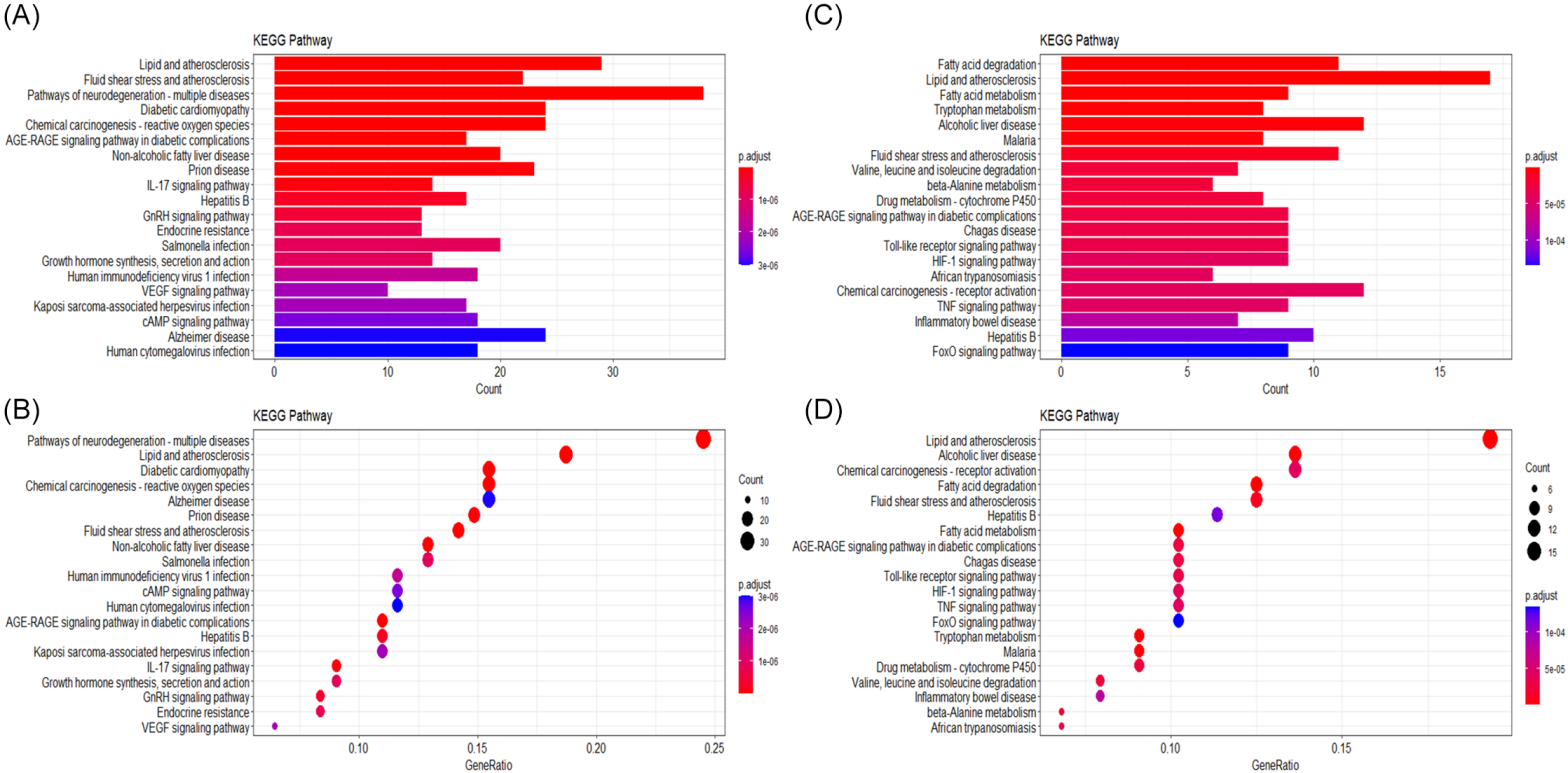
KEGG analysis of upregulated and downregulated DEOSGs. (A, B) The top 20 KEGG enrichment terms of upregulated DEOSGs. (B, C) The top 20 KEGG enrichment terms of downregulated DEOSGs.

Figure 1 shows the workflow used for bioinformatics analysis of the datasets. We first identified 544 OS genes by taking the intersecting three datasets (Figure 2A). Then, we analyzed the expression of 544 OS genes in 50 normal samples and 374 tumor tissues from the TCGA dataset. There were 282 OS genes identified as DEOSGs, including 93 down‐regulated genes and 189 up‐regulated genes that fulfilled the screening criteria (FDR <0.05 and |Log2FC| ≥ 0.5). In Figure 2B,C, we illustrated the distribution of DEOSGs expression in HCC. Figures 3 and 4 present the analytical results of molecular mechanisms and potential pathways of the DEOSGs using the GO and KEGG analysis.

### 4.2 Screening of DEOSGs associated with prognosis and development of risk signatures

**FIGURE 5:**
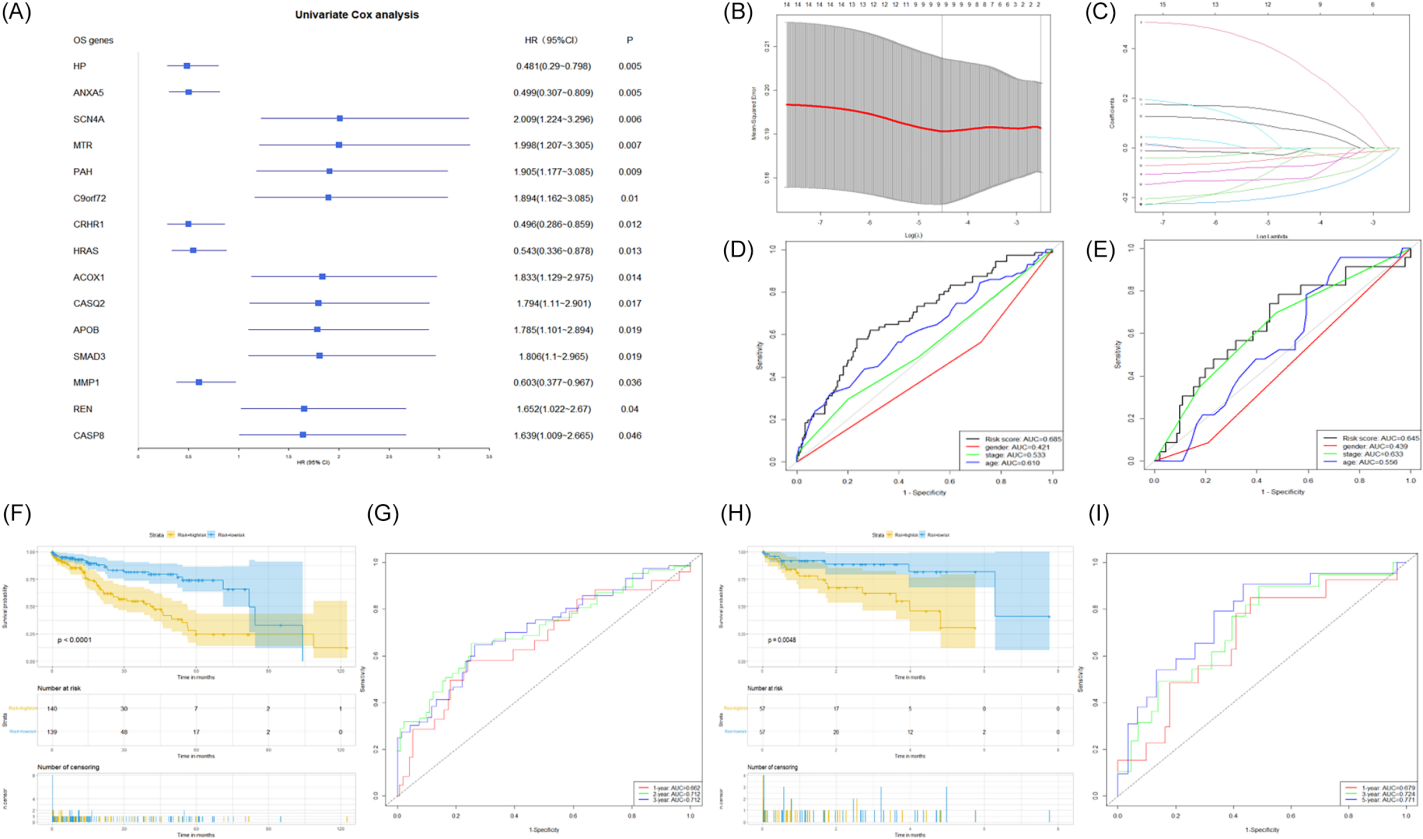
Construction of risk signature in the TCGA and GEO datasets. (A) Univariate Cox regression analysis for identification of prognosis‐associated DEOSGs. (B, C) The number of constructed prognostic risk signature genes was determined by LASSO analysis. (D, E) The ROCs and AUCs of risk score and clinical‐pathological factors, including age, gender, and stage, in TCGA and GEO datasets. (F, H) KM survival curves of TCGA and GEO datasets. (G, I) TimeROC curves for 1, 2, and 3‐year overall survival in TCGA and GEO datasets.

**FIGURE 6:**
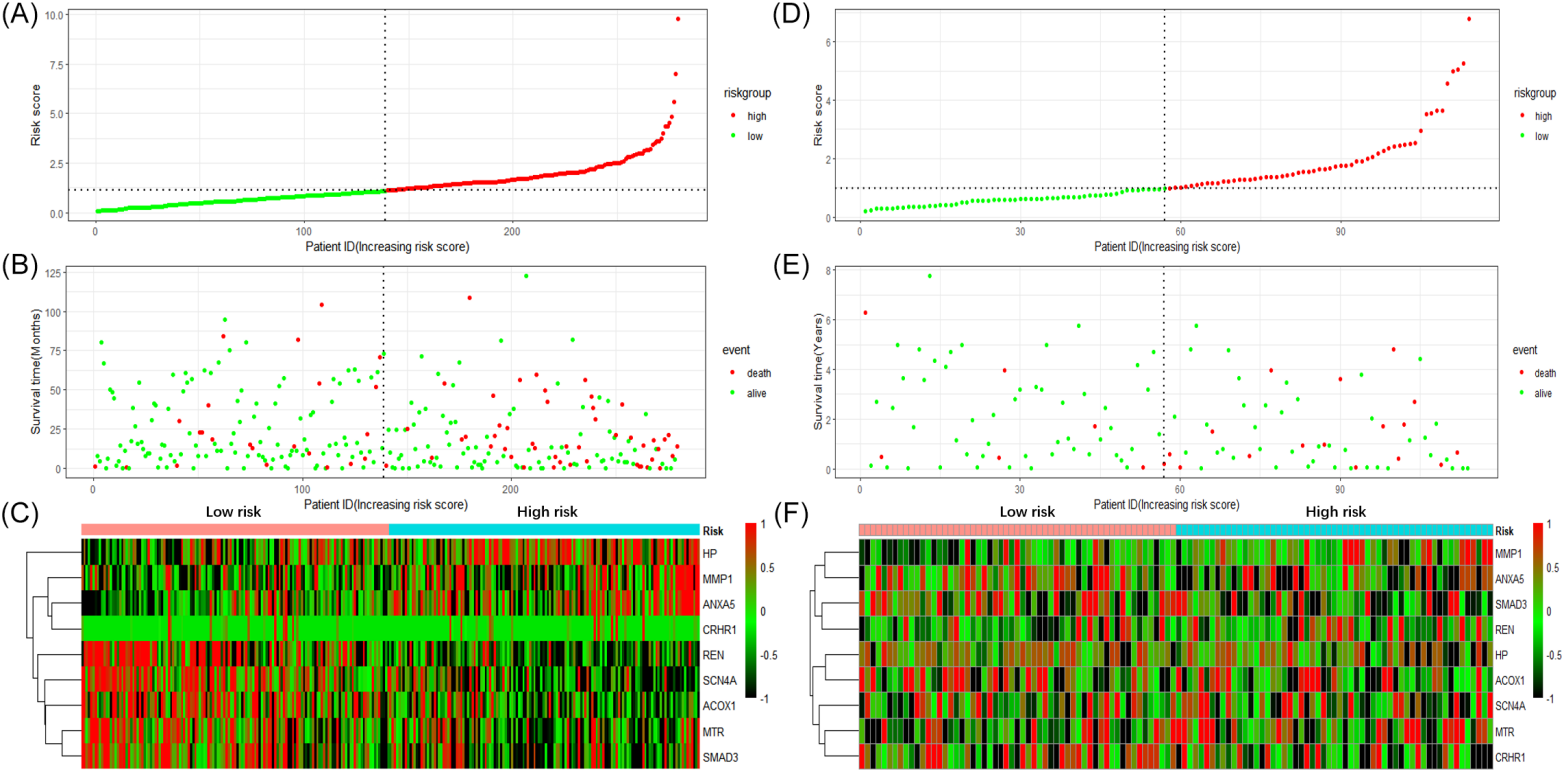
Association of risk score with survival time and prognosis‐related OS gene expression. (A, D) The risk curves of risk score distribution in the TCGA and GEO datasets. (B, E) Scatterplot of survival status of HCC patients in the TCGA and GEO datasets. (C, F) The heatmap displayed the expression levels of prognostic‐related DEOSGs in the high‐risk and low‐risk groups in the TCGA and GEO datasets.

To discover prognosis‐associated DEOSGs, KM survival and univariate Cox analyses were used to analyze 15 DEOSGs, and 15 DEOSGs were chosen as HCC prognosis‐associated candidate OS genes with *p* < .05. (Figure 5A). Subsequently, the LASSO algorithm was used to narrow down the range of prognosis‐related OS genes, and 9 DEOSGs were selected to construct the risk signature of OS genes (Figure 5B,C). The coefficients of the 9 DEOSGs are shown in Table 1. Based on the median risk score, HCC patients in two datasets were separated into low‐risk and high‐risk categories. (Figure 6A–F). The survival ROC curves of the risk signature in the TCGA and GEO were 0.685 and 0.645, respectively (Figure 5D,E). The results showed a better performance of the risk signature than clinical‐pathological factors in both datasets. Overall survival was considerably worse in high‐risk HCC patients than in low‐risk patients in the TCGA and GEO datasets. (Figure 5F,H). We also plot the time‐dependent ROC of risk signatures in the two datasets. The AUCs of 1, 2, 3‐year were 0.662, 0.712.0.712 in TCGA dataset, and 0.679, 0.724, 0.771 in GEO dataset, respectively (Figure 5G,I). All the above results demonstrated that risk signatures have moderate specificity and sensitivity.

**TABLE 1:** Nine prognosis‐associated OS genes in the TCGA dataset were identified by univariate Cox analysis and LASSO analysis.

OS genes	Univariate Cox regression analysis				LASSO coefficient
	*P* value	HR	Lower 95% CI	Upper 95% CI	
SCN4A	.006	2.009	1.224	3.296	−0.031
HP	.005	0.481	0.290	0.798	0.023
MTR	.007	1.998	1.207	3.305	−0.008
ACOX1	.014	1.833	1.129	2.975	−0.013
MMP1	.036	0.603	0.377	0.967	0.006
SMAD3	.019	1.806	1.100	2.965	−0.033
REN	.040	1.652	1.022	2.670	−0.011
ANXA5	.005	0.499	0.307	0.809	0.022
CRHR1	.012	0.496	0.286	0.859	0.030

However, when HCC patients were divided into living and dead groups, growing risk scores were no longer significantly associated with HCC patient survival time. (Figure 6B,E), suggesting that our risk signature only predicts survival in the entire population and cannot be utilized to predict HCC patient survival time.

**FIGURE 7:**
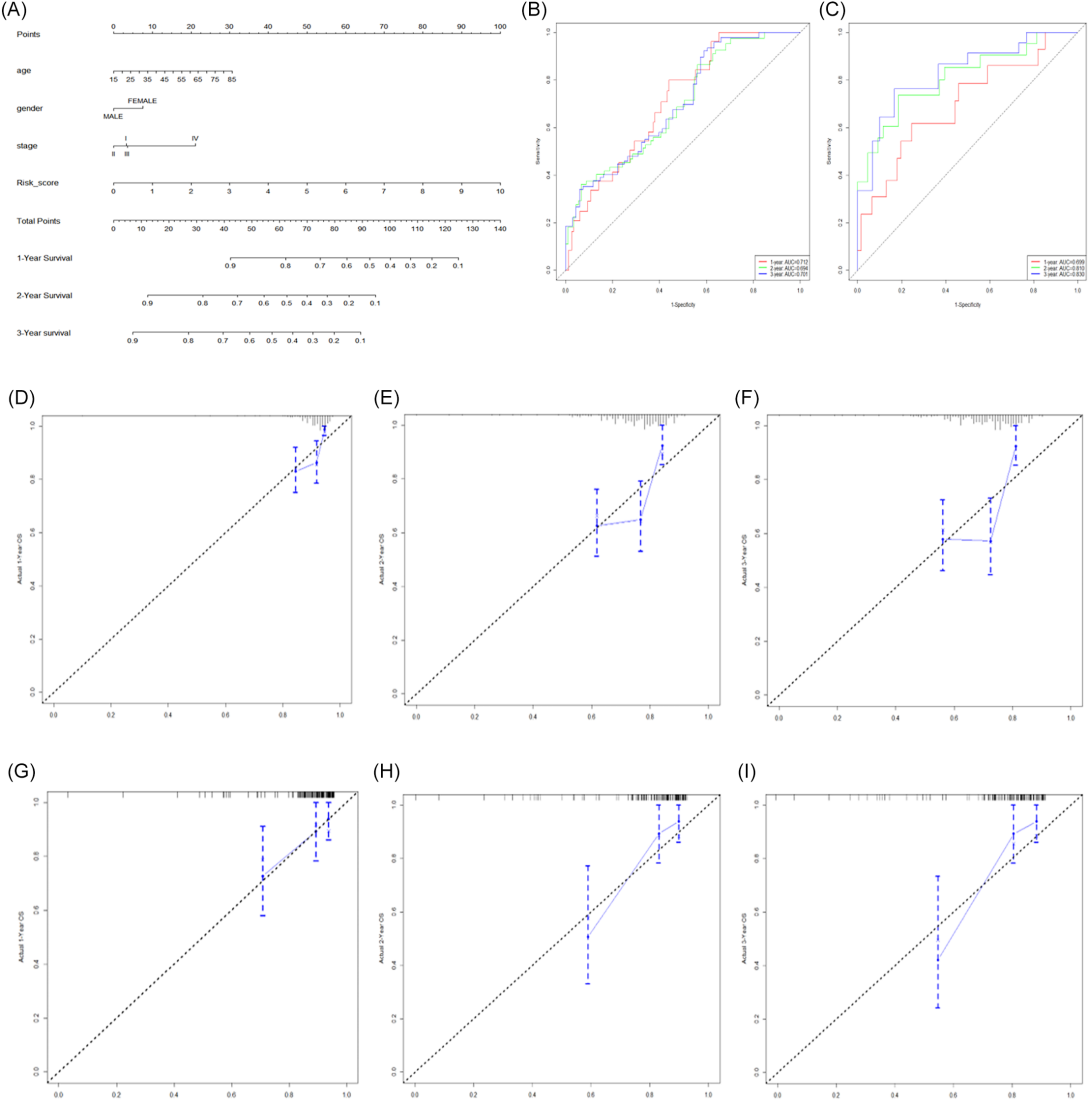
Construction and validation of prognostic nomogram. (A) The prognostic nomogram for HCC patients. (B, C) The time‐dependent ROC of nomogram at 1, 2, and 3 years in the TCGA and GEO datasets. (D–F) The 1,2,3‐years calibration curves of nomogram in the TCGA dataset. (G–I) The 1,2,3‐years calibration curves of nomogram in the GEO dataset.

Furthermore, Univariate and multivariate Cox regression analyses were performed to see whether the risk signature was a prognostic characteristic. The risk signature was an independent predictive characteristic in the TCGA dataset that was substantially linked with the prognosis of HCC. The nomogram is a quantitative model that is used to forecast clinical outcomes in HCC patients. Following that, we created a predictive nomogram for HCC patients using the TCGA dataset, based on risk signature and clinical‐pathological variables (Figure 7A). We further evaluated the predictive performance of this nomogram in the TCGA and GEO datasets using time‐dependent ROC and calibration curves. The AUCs of the nomogram at 1, 2, and 3 years were 0.712, 0.694, and 0.701 in the TCGA dataset (Figure 7B), and 0.699, 0.810, and 0.830 in the GEO dataset (Figure 7C). The calibration curves in the TCGA (Figure 7D–F) and GEO datasets (Figure 7G–I) demonstrated that the nomogram had powerful calibration performance.

### 4.3 Evaluating the levels of expression of prognostically relevant DEOSGs in HCC patients

**FIGURE 8:**
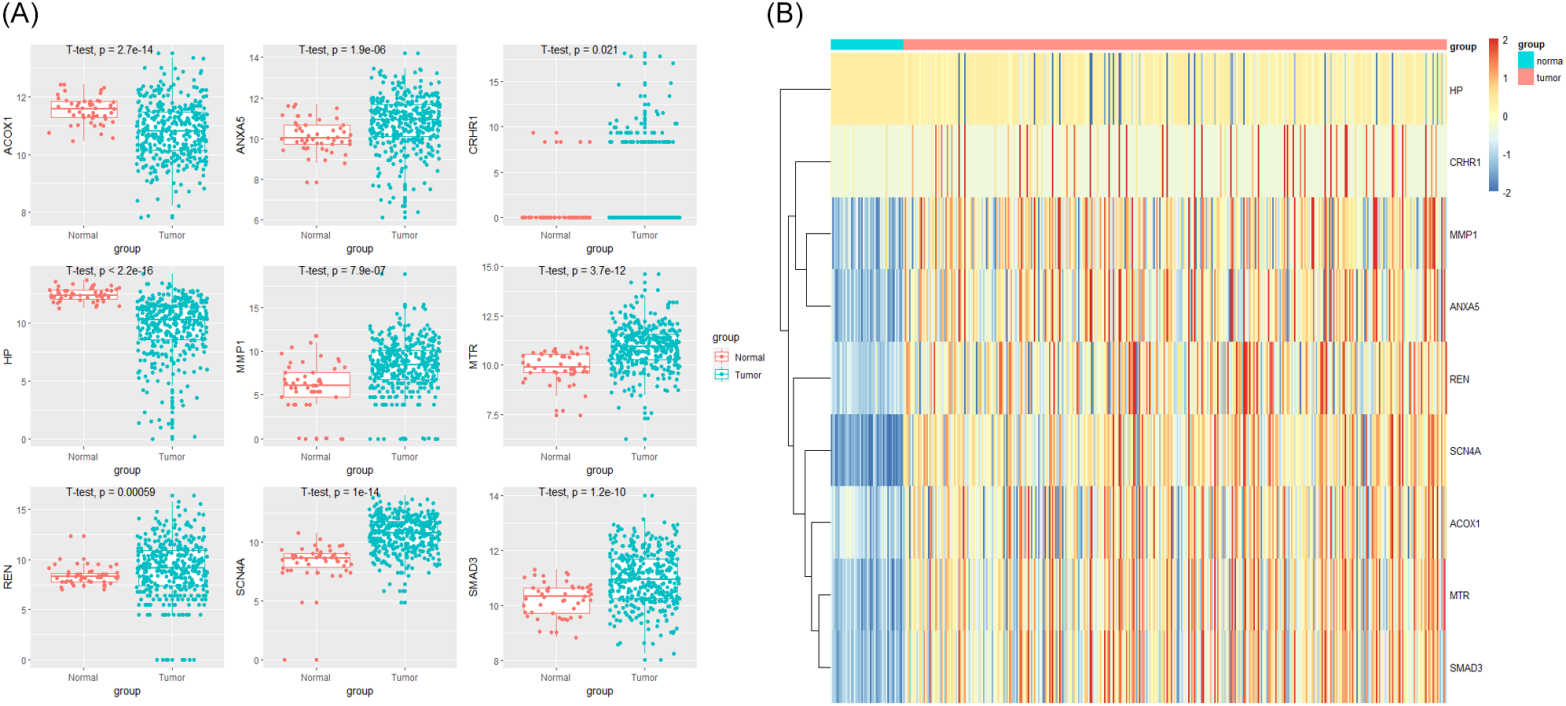
Expression level of prognosis‐related DEOSGs. (A) The box plot of prognostic‐related DEOSGs in the TCGA dataset. (B) The heatmap of prognostic‐related DEOSGs in the TCGA dataset.

We extracted the expression values of each prognostic‐related OS gene from the TCGA dataset and further to investigate the transcript levels of DEOSGs in HCC patients. The TCGA database was used to extract the expression values of each important gene, and box plots (Figure 8A) and heat maps (Figure 8B) were created.

### 4.4 Screening of DEOSGs related small‐molecule drugs

**FIGURE 9:**
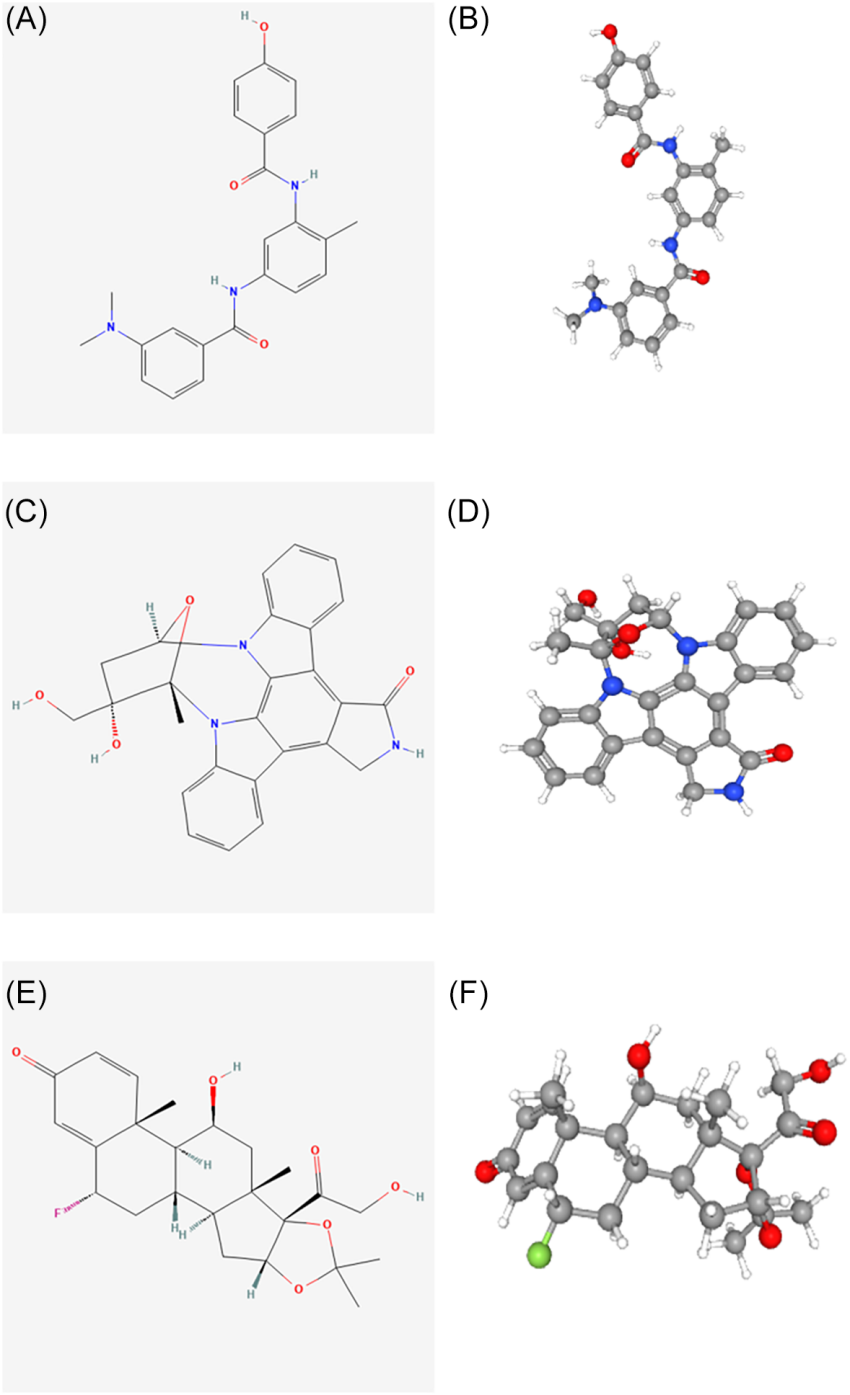
Structures of the screened small‐molecule drugs. (A, B) 2D and 3D structures of ZM‐336372. (C, D) 2D and 3D structures of lestaurtinib. (E, F) 2D and 3D structures of flunisolide.

To find prospective medications for the treatment of HCC, we uploaded up‐ and down‐regulated DEOSGs to the L1000FWD website and matched them with small‐molecule therapeutic methods. Table 2 summarizes the top 10 small‐molecule drugs, as well as their similarity scores. The top three drugs were ZM‐336372, lestaurtinib, and flunisolide, all of which were predicted to have an inverse effect on DEOSGs expression. Using the PubChem website, they were visualized in 2D and 3D forms (Figure 9A–F). These potential small molecule drugs are expected to inhibit OS‐induced gene expression and pave the way for the development of targeted treatments for the treatment of HCC.

**TABLE 2:** The screened drugs for HCC treatment.

Drug	Similarity score	*p*‐value	q‐value	Z‐score	Combined score	MOA	Predicted MOA
ZM‐336372	−0.0833	2.6E‐09	0.0000453	1.81	−15.57	RAF inhibitor	MEK inhibitor
Lestaurtinib	−0.0758	3.24E‐07	0.00139	1.66	−10.78	FLT3 inhibitor, growth factor receptor inhibitor, JAK inhibitor	adrenergic receptor antagonist
Flunisolide	−0.0758	1.22E‐07	0.000747	1.83	−12.66	Cytochrome P450 inhibitor	glucocorticoid receptor agonist
Emetine	−0.072	6.49E‐07	0.00197	1.78	−11.03	Protein synthesis inhibitor	HDAC inhibitor
BRD‐K86574132	−0.072	1.5E‐07	0.000805	1.86	−12.68	Unknown	PI3K inhibitor
Ribavirin	−0.072	8.56E‐07	0.00204	1.74	−10.57	Antiviral	Protein synthesis inhibitor
L‐690488	−0.072	2.99E‐07	0.00139	1.81	−11.78	Unknown	Retinoid receptor agonist
BRD‐K03176945	−0.072	9.96E‐07	0.00213	1.72	−10.31	Unknown	Cyclooxygenase inhibitor
Fluticasone	−0.0682	1.56E‐06	0.00257	1.85	−10.76	Unknown	Glucocorticoid receptor agonist
Azacitidine	−0.0682	2.05E‐06	0.00302	1.67	−9.52	DNA methyltransferase inhibitor	HDAC inhibitor

## 5 DISCUSSION

Current research on the pathogenesis of HCC has concentrated on the cellular and molecular levels. However, few studies have attempted to utilize the research results in clinical situations. In our study, 282 DEOSGs were identified based on the TCGA dataset, and GO and KEGG enrichment analyses were performed. The results of KEGG enrichment analysis revealed that DEOSGs were remarkably related to lipid and atherosclerosis, fatty acid degradation, ROS, and hepatitis B. Additionally, DEOSGs were found to be considerably concentrated in a variety of biological processes, including response to oxidative stress, cellular response to chemical stress, response to xenobiotic stimulus, and fatty acid metabolic process. All of these biological processes are linked to the onset and progression of liver disease.^18^, ^19^

To discover OS genes associated with prognosis, we used univariate cox analysis and LASSO analysis, generating 9 DEOSGs: ANXA5, MMP1, MTR, REN, SCN4A, HP, ACOX1, and SMAD3.

We also analyzed the levels of expression of these genes in the TCGA dataset as well as the levels of protein expression in the HPA database. The results showed that ANXA5, MMP1, MTR, REN, SCN4A, and SMAD3 were upregulated in HC samples, while HP and ACOX1 were downregulated in tumor tissues. The results of this study were the same as those of previous studies. Overexpression of ANXA5 and MMP1 in HCC may enhance clinical progression and lymphatic metastasis.^20^, ^21^ Moreover, low levels of HP expression are related to superior HCC tumor differentiation and enhanced five‐year overall survival in HCC patients.^22^

To evaluate whether the risk signature of all these specific DEOSGs can be used as a prognostic marker, we performed univariate and multivariate Cox regression analyses, which revealed that this risk signature is an independent predictor with a strong predictive significance for HCC. Survival and ROC analysis confirmed the prognostic value of risk signature for predicting HCC. Furthermore, the nomogram analysis revealed that risk characteristics were of significant value in predicting overall survival in HCC patients, and their predictive power was significantly higher than that of other clinicopathological characteristics. Thus, OS plays a pivotal role in all stages of carcinogenesis and cancer progression.^23^, ^24^

This study has several advantages. On one hand, the risk signature was performed external validation, demonstrating the robustness and reliability of the risk signature. On the other hand, a nomogram for quantitative calculations was further established, which facilitates clinical dissemination and application. The present study still has some limitations. First, this study is a retrospective analysis requiring further validation by prospective studies. Second, the mechanism of these genes and HCC progression needs to be further explored.

In conclusion, our study revealed 9 DEOSGs associated with overall survival in HCC and created a prognosis‐related risk signature and nomogram based on these 9 DEOSGs. Simultaneously, we screened small molecule drugs that could be linked to DEOSGs. This study sheds new light on the pathogenesis of HCC as well as potential therapeutic approaches for HCC.

## AUTHOR CONTRIBUTIONS

**Guole Nie:** Formal analysis (lead); validation (lead); visualization (lead); writing – original draft (lead); writing – review and editing (lead). **Xingwang Zhu:** Conceptualization (equal); writing – original draft (equal). **Honglong Zhang:** Investigation (supporting); resources (supporting). **Haiping Wang:** Formal analysis (supporting); resources (supporting); software (supporting). **Jun Yan:** Project administration (supporting); supervision (supporting); writing – review and editing (supporting). **Xun Li:** Project administration (lead); writing – review and editing (supporting).

## FUNDING INFORMATION

None.

## CONFLICT OF INTEREST STATEMENT

The authors state that no commercial or financial connections that might be considered as a possible conflict of interest existed during the research.

## ETHICAL STATEMENT

The study does not contain any experiments on humans or animals, and/or the use of human tissue samples performed by any of the authors. Medical ethical approval and informed permission were waived for the analysis of data not recognized in the SEER database.

## Data Availability

The datasets for this study can be found in the TCGA (https://portal.gdc.cancer.gov/) and GEO (https://www.ncbi.nlm.nih.gov/geo/) database.
